# Sleep quality and its correlates in people with epilepsy: A multicenter cross‐sectional study in Germany

**DOI:** 10.1111/epi.18599

**Published:** 2025-08-22

**Authors:** Johann Philipp Zöllner, Catrin Mann, Laurent Willems, Felix von Podewils, Lisa Langenbruch, Laura Bierhansl, Susanne Knake, Katja Menzler, Juliane Schulz, Bernadette Gaida, Felix Rosenow, Adam Strzelczyk

**Affiliations:** ^1^ Goethe University Frankfurt, Epilepsy Center Frankfurt Rhine‐Main, Department of Neurology University Medicine Frankfurt Frankfurt am Main Germany; ^2^ Goethe University Frankfurt, Center for Personalized Translational Epilepsy Research (CePTER) Frankfurt am Main Germany; ^3^ Epilepsy Center, Department of Neurology University Hospital Greifswald Greifswald Germany; ^4^ Epilepsy Center Münster‐Osnabrück, Department of Neurology with Institute of Translational Neurology University of Münster Münster Germany; ^5^ Department of Neurology Klinikum Osnabrück Osnabrück Germany; ^6^ Department of Neurology University Medical Center Schleswig‐Holstein, Campus Lübeck Lübeck Germany; ^7^ Epilepsy Center Hessen, Department of Neurology Philipps‐University Marburg Marburg Germany

**Keywords:** affective disorders, anxiety, depression, quality of life, seizures

## Abstract

**Objective:**

Sleep disturbances and epilepsy are closely interrelated. This study aimed to examine associations between sleep quality, affective symptoms, and quality of life (QoL) in people with epilepsy (PWE), and to identify clinical and sociodemographic factors linked to impaired sleep.

**Methods:**

We conducted a multicenter cross‐sectional study of adult PWE across four tertiary epilepsy centers in Germany. We assessed sleep quality using the Pittsburgh Sleep Quality Index (PSQI), affective symptoms with the Hospital Anxiety and Depression Scale (HADS), and epilepsy‐specific QoL with the 31‐item Quality of Life in Epilepsy (QOLIE‐31) inventory. We evaluated sociodemographic and clinical predictors of sleep quality and QoL using univariable and multivariable analyses. Comparisons were made with normative data from the general German population and individuals with chronic migraine.

**Results:**

Of 449 individuals (mean age 39.9 years, 58.1% women), 221 (49.2%) were identified as “poor sleepers,” significantly exceeding rates in the general population (35.9%, *p* < .001). In multivariable analysis, poor sleep quality was independently associated with female sex (odds ratio [OR] 2.02, 95% confidence interval [CI] 1.36–3.01, *p* = .001), unemployment (OR 1.74, 95% CI = 1.10–2.73, *p* = .017), anxiety (OR = 3.79, 95% CI = 2.39–6.04, *p* < .001), and depression (OR = 2.19, 95% CI = 1.36–3.54, *p* = .001). In addition, daily seizures were linked to worse sleep quality (OR = 2.55, 95% CI = 1.03–6.30, *p* = .042). Poor sleep was independently associated with lower epilepsy‐related QoL after adjusting for affective symptoms and seizure frequency (OR = 1.79, 95% CI = 1.20–2.68, *p* = .005).

**Significance:**

Sleep quality in PWE is significantly impaired and strongly associated with anxiety, depression, and sociodemographic variables in addition to epilepsy‐specific factors. Poor sleep independently correlates with diminished QoL, supporting the value of routine screening for affective symptoms and sleep disturbances in epilepsy care.

**Key points**

- People with epilepsy (PWE; mean ± standard deviation [SD] Pittsburgh Sleep Quality Index [PSQI] score 6.5 ± 3.8) reported worse sleep quality than the general population (5.0 ± 3.4), mainly for sleep disturbance and daytime dysfunction (all *p*'s < .001).
- Poor sleep was strongly linked to anxiety (odds ratio [OR] 3.8, *p* < .001), depression (2.2, *p* = .001), female sex (2, *p* = .001), and daily seizures (2.6, *p* = .042).
- Poor sleep quality was independently linked to lower epilepsy‐specific quality of life (QoL; OR 1.8, 95% confidence interval 1.2‐2.7, *p* = .005), after adjusting for mood and seizure frequency.
- These findings support screening for sleep and mood issues in epilepsy care, especially for those with pharmacoresistant epilepsy.
- Longitudinal and interventional studies are needed to clarify the direction and modifiability of these associations.

## 1 INTRODUCTION

Epilepsy is characterized by spontaneous recurrent seizures. It is one of the most common chronic neurological disorders, affecting millions of individuals worldwide.^1^ In Germany, ~1% of the population has epilepsy, and at least 10% will have one seizure in their lifetime.^2^ A relevant yet often overlooked aspect of epilepsy management is the interplay between epilepsy and sleep.^3^, ^4^ As a fundamental physiological process, sleep is critical in maintaining various brain functions, including memory consolidation, mood regulation, and cognitive performance.^3^ However, people with epilepsy (PWE) often experience sleep disturbances, which has been reported to affect their overall quality of life (QoL).^2^, ^5^, ^6^

The relationship between epilepsy and sleep is multifaceted and reciprocal. Sleep deprivation is a major seizure trigger, especially in those with genetic generalized epilepsies, which also commonly manifest after waking (e.g., juvenile myoclonic epilepsy). Focal epileptiform discharges are commonly activated during non–rapid eye movement sleep, and certain focal seizures, most prominently hypermotor seizures in frontal lobe epilepsies, often occur nocturnally during sleep Stage 2.^3^, ^7^ Epilepsy is also likely associated with sleep‐disordered breathing.^8^ These features of epilepsy can again reduce sleep architecture and, thus, sleep efficiency and quality, sometimes leading to vicious cycles of seizure worsening. Notably, sudden unexpected death in epilepsy (SUDEP) occurs mostly during sleep, usually after bilateral tonic–clonic seizures, and is associated with an annual mortality rate of 1:1000 in PWE.^3^, ^9^ In addition, antiseizure medications (ASMs), the essential cornerstone of epilepsy treatment, can have variable effects on sleep, further complicating this relationship. Although some ASMs may improve sleep by reducing seizure activity, others may adversely affect sleep quality, leading to daytime sleepiness or insomnia.^3^

Understanding the links between epilepsy, sleep, and cognitive and affective functioning is essential to improving patient outcomes. Despite the significant impact on patient well‐being, few comprehensive studies were able to thoroughly examine the interaction between sleep quality, seizures, and further clinical variables such as age, sex, depression, and anxiety in large PWE cohorts. A recent meta‐analysis could not reveal specific predictors of impaired sleep quality in PWE, potentially due to study heterogeneity.^10^

This study aimed to address this gap by evaluating the impact of epilepsy‐associated variables on sleep quality in PWE and the effect of sleep quality on epilepsy‐related QoL, using data from a large German survey. By analyzing sleep quality and its associations with QoL and mental health, it aimed to provide insights that could inform better management strategies for epilepsy, ultimately improving the lives of PWE and their families.

## 2 METHODS

### 2.1 Study settings, patients, and design

The multicenter, cross‐sectional Epi2020 study was conducted at four different epilepsy centers in Germany (Frankfurt am Main, Greifswald, Marburg, and Münster). These centers all offer specialized inpatient and outpatient care for PWE, including individuals with developmental and/or epileptic encephalopathies (DEEs) and epilepsy‐associated syndromes.^11^, ^12^, ^13^ Patients were instructed to complete a standardized questionnaire that focused on their QoL and other health care–related aspects of epilepsy. Epilepsy type was captured as focal epilepsy with lobe definition, if available (e.g., “temporal lobe epilepsy”), genetic generalized epilepsy (=idiopathic generalized epilepsy), or “unclassified.” All adults who had a confirmed epilepsy diagnosis between October 2020 and December 2020 were eligible to participate. All questionnaires designed as self‐report instruments (e.g., the Pittsburgh Sleep Quality Index [PSQI]) were to be completed by the PWE themselves. Proxy assistance in completing the questionnaires was permitted only in cases of motor impairment, ensuring that the data reflect the intended self‐reported format. Written informed consent was mandatory before enrollment.

The Epi2020 study was registered with the German Clinical Trials Register (DRKS00022024; Universal Trial Number:) and was approved by the ethics committee of the Goethe‐University Frankfurt (reference number 19‐440). It adhered to the Strengthening the Reporting of Observational Studies in Epidemiology (STROBE) and Reporting of studies Conducted using Observational Routinely collected health Data (RECORD) guidelines.^14^, ^15^ It compared selected sleep and affective/mood scores of PWE with normative values from the German general population^16^, ^17^ and with those of individuals with episodic migraine, another common chronic neurologic condition with intermittent episodes of heightened clinical severity.^18^

### 2.2 Scores and metrics

The Pittsburgh Sleep Quality Index (or PSQI) is a widely utilized self‐report instrument designed to assess sleep quality and disturbances over 1 month. It consists of 19 items assessing seven domains: subjective sleep quality, sleep latency, sleep duration, habitual sleep efficiency, sleep disturbances, use of sleeping medications, and daytime dysfunction. These domain scores are summed to produce a global score ranging from 0 to 21, with higher scores indicating worse sleep quality. A global PSQI score >5 is typically used to identify individuals with significant sleep disturbances. Interpreting PSQI scores provides a comprehensive overview of sleep quality, allowing for the identification of sleep‐related issues that may impact overall health and well‐being. According to the original author's recommendations, we included only those individuals who fully completed the PSQI instrument in the final analysis.^19^

The 31‐item health‐related Quality of Life in Epilepsy (QOLIE‐31) inventory is a well‐established instrument and is considered the gold standard for assessing health‐related QoL in PWE.^20^, ^21^ It consists of 30 questions and a visual analog scale covering seven subcategories: worry about seizures, general QoL, emotional well‐being, energy and fatigue, cognitive impairment, medication effects, and social function. We analyzed the QOLIE‐31 according to the recommendations of the QOLIE development group to determine the total score and the T‐score.^22^

The Hospital Anxiety and Depression Scale (HADS) was also administered, a well‐established self‐report instrument for screening anxiety and depression symptoms with high utility in clinical practice. The total HADS score can be used, or it can be divided into individual subscales for depression (HADS‐D) and anxiety (HADS‐A). A subscale score of ≥8 was considered indicative of depression or an anxiety disorder.^23^, ^24^

Several demographic and disorder‐related factors were correlated with the measured items and scores. Participants reported their sex, age, body mass index (BMI), seizure frequency, type and dose of ASM, type and dose of non‐ASM sleep‐related medications (e.g., antidepressants, neuroleptics, antihistaminergic or melatoninergic drugs, or herbal remedies), comorbid conditions and medications, relationship status, employment status, and the presence of a disability certificate. The treating physician provided additional information on the type and duration of epilepsy.

### 2.3 Statistical analysis

Data input, statistical workup, and graphical representation were performed using SPSS (version 27 or higher; IBM Corp., Armonk, NY, USA) and the R statistical software (version 4.2.2; R Core Team, Vienna, Austria). A *p*‐value of <.05 (two‐sided) was considered statistically significant.

Descriptive analyses were conducted for clinical and sociodemographic characteristics and the evaluated instruments. We compared PSQI and HADS‐D scores with normative values from the general German population using *t*‐tests, as only aggregate mean and standard deviations (SDs) were available from the literature.^16^, ^17^

Univariable analyses were conducted using the chi‐square or Kruskal–Wallis tests, depending on the data measurement level. Multivariable ordinal (ordered logit) regression analyses were conducted with PSQI as the dependent variable and factors significant in the univariable analyses or selected based on prior knowledge from the literature as independent variables, while checking for multicollinearity.^16^, ^17^ The variables age, sex, BMI, seizure frequency, epilepsy type, relationship status, occupational situation, HADS‐D, HADS‐A, disability certificate status, and use of any sleep‐related medication/sedatives were included in the multivariable model. Multivariable analyses with total QOLIE‐31 scores as the dependent variable were performed similarly, including all variables listed previously and the PSQI score. Correlations between scores were assessed using Kendall's rank correlation coefficient (*τ*).

## 3 RESULTS

### 3.1 Sociodemographic characteristics

This study included 486 adult PWE. Of these, only the 449 (92.4% of all participants) who completed the PSQI were included in the final study population per the guidelines for evaluating the PSQI instrument.^19^ The participants had a mean age of 39.9 ± 15.3 years (18–83), and 261 (58.1%) were women (Table 1).

**TABLE 1:** Participants' clinical and sociodemographic characteristics (*n* = 449).

Age, years	
Mean ± SD [range]	39.9 ± 15.3 [18–83]
Sex, *n* (%)	
Men	188 (41.9)
Women	261 (58.1)
Body mass index (BMI)	
Mean ± SD [range]	25.7 ± 5.5 [14.4–64.5]
At least severe obesity, BMI ≥35 kg/m^2^, *n* (%)	61 (13.6)
Epilepsy type, *n* (%)	
Focal: Frontal lobe epilepsy	37 (8.2)
Focal: Other lobes	264 (58.8)
Genetic generalized epilepsy	97 (21.6)
Unclassified	51 (11.4)
Epilepsy onset (years; *n* = 434)	
Mean ± SD [range]	24.4 ± 16.1 [0–79]
Mean duration of epilepsy (years; *n* = 426)^a^	
Mean ± SD [range]	16.6 ± 14.6 [0–70]
Seizure frequency, *n* (%)	
Daily	19 (4.2)
Weekly	43 (9.6)
Monthly	78 (17.4)
Once every 6 months	41 (9.1)
Once per year	49 (10.9)
No seizures in >1 year	194 (43.2)
Not reported	25 (5.6)
Number of anti‐seizure medications (ASMs), *n* (%)	
0	16 (36.0)
1	186 (41.4)
2	162 (36.1)
≥3	85 (18.9)
Mean ± SD [range]	1.7 ± .9 [0–6]
Relationship status, *n* (%)	
Married/in a relationship	253 (56.3)
Divorced/separated	21 (4.7)
Single/living with relatives	76 (16.9)
Living alone	85 (18.9)
Widowed	5 (1.1)
Not reported	9 (2.0)
Occupational situation, *n* (%)	
Working	243 (54.1)
Stay‐at‐home	22 (4.9)
Vocational training	42 (9.4)
Unemployed	33 (7.3)
Disability pension	68 (15.1)
Retired	34 (7.6)
Other or n/a	7 (1.6)
Certificate of disability, *n* (%)	
No	191 (42.5)
Yes	256 (57.0)
Not reported	2 (.4)
Patients taking antidepressant medication, *n* (%)	
SSRI/S(S)NRI (citalopram, escitalopram, fluoxetine, duloxetine, sertraline, venlafaxine)	28 (6.2)
TCA (amitriptyline, mirtazapine)	6 (1.3)
Melatoninergic (agomelatine, melatonin)	3 (.7)
Any antidepressant^b^	36 (8)
Patients taking sleep medication, *n* (%)	
Z‐Drugs (i.e., zopiclone)	1 (.2)
Antihistaminergic (promethazine)	1 (.2)
Other (*Lavendula* oil)	1 (.2)
Taking antipsychotic medication, *n* (%)	
Amisulpride, risperidone, prothipendyl, quetiapine, or pipamperone	9 (2.0)
Taking chronic benzodiazepines, *n* (%)	
Clonazepam, clobazam, bromazepam, or diazepam	9 (2.0)
Taking barbiturates, *n* (%)	
Primidone, phenobarbital	12 (2.7)
Any sleep‐related medication/sedatives^b^	60 (13.4)
Self‐reported visit to psychiatrist/psychotherapist, *n* (%)	
Yes, within past 3 months	29 (6.5)

Abbreviations: SD, standard deviation; SSRI, selective serotonin reuptake inhibitor; S(S)NRI, selective serotonin‐norepinephrine reuptake inhibitor; TCA, tricyclic antidepressant.

^a^ From the first seizure.

^b^ More than one medication class is possible per individual.

### 3.2 Clinical characteristics

Focal epilepsy was present in 301 participants (67.0%), of which 37 had frontal lobe epilepsy, which is associated with frequent nocturnal seizures. Genetic generalized epilepsy was present in 97 participants (21.6%). Epilepsy was present for a mean time of 16.6 years (SD ± 14.6, range 0–70), and the participants were treated with a mean of 1.7 ASM (SD ± .9, median 1, range 0–6). Current seizure freedom (>1 year) was achieved in 194 participants (43.2%). Outpatient treatment by a psychiatrist/psychotherapist (i.e., self‐reported visit within the past 3 months) was being provided to 29 participants (6.5%).

### 3.3 Medication use

Non‐ASM sleep‐related, chronic benzodiazepine or barbiturate medication (sedatives) was used by 60 participants (13.4%). Daridorexant, suvorexant, lemborexant, diphenhydramine, doxylamine, and zolpidem were not used by any participants. Given the likely substantial differences in the indications for acute vs chronic benzodiazepine use, and the inability of the questionnaire to distinguish between these patterns, only chronic benzodiazepine use was considered in this analysis.

### 3.4 PSQI

**FIGURE 1:**
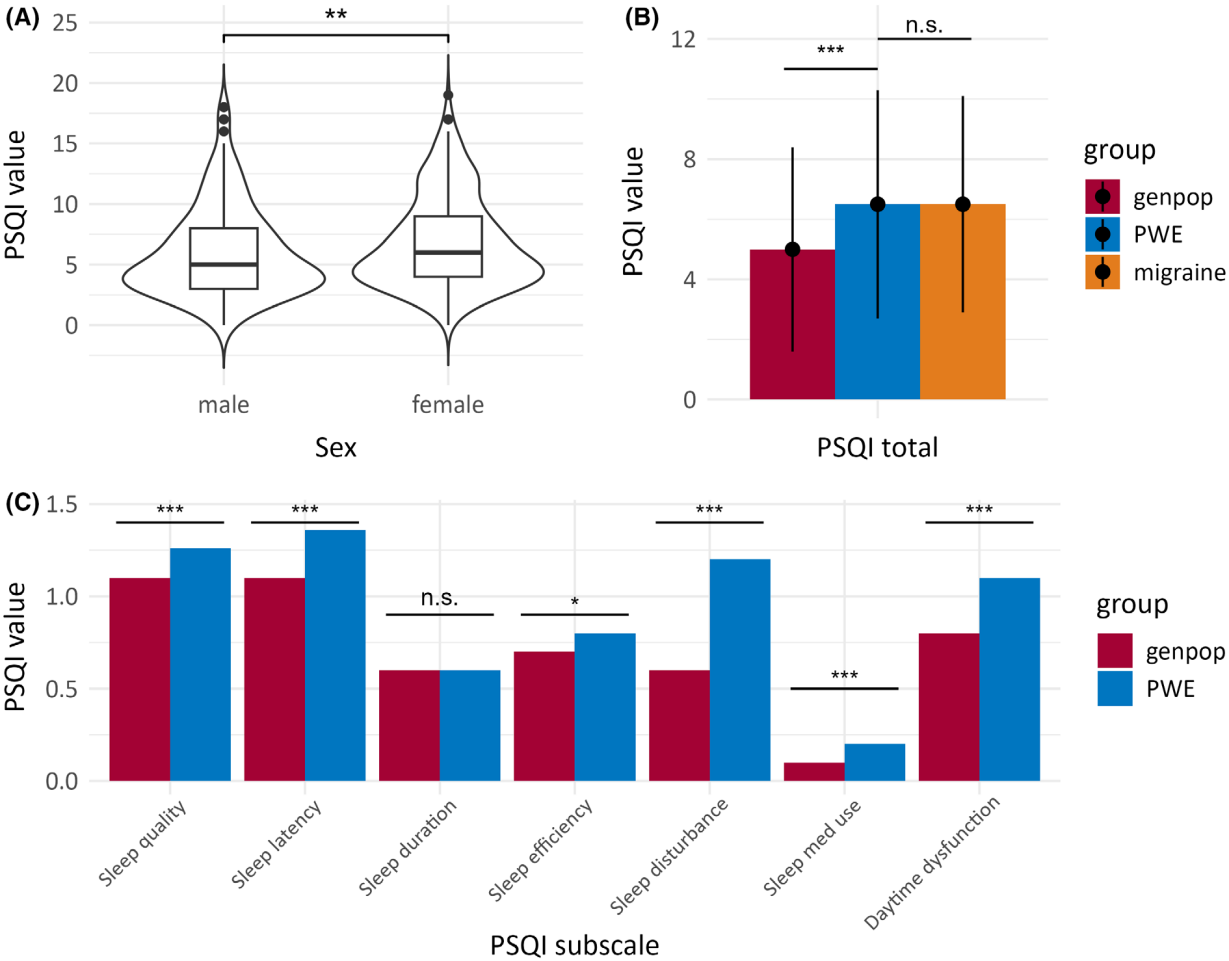
Sleep quality assessed by the Pittsburgh Sleep Quality Index (PSQI) instrument in people with epilepsy (PWE), compared to the German general population and individuals with migraine. (A) Women with epilepsy had worse sleep quality than men (mean 6.8 [standard deviation (SD) ± 3.8] vs 6.0 [SD ± 3.7], *p* = .009), a common finding on the PSQI instrument that is also seen in the general German population (genpop). (B) Overall sleep quality was significantly worse in PWE than in the genpop (*p* < .0001), but did not differ significantly from people with chronic migraine. (C) The largest absolute differences in the PSQI subscales were evident for increased sleep disturbances, daytime dysfunction, and sleep latency, but only self‐reported sleep duration was not worse in PWE than in the genpop. n.s., not significant. Significance levels: *, *p* < 0.05; **, *p* < 0.01; ***, *p* < 0.001.

The mean PSQI score in the PWE cohort was 6.5 (SD ± 3.8), with a median of 5 points. Women had worse sleep quality than men (mean 6.8 [SD ± 3.8] vs 6.0 [SD ± 3.7], *p* = .009), a common finding in the PSQI instrument and also seen in the general German population (Figure 1 and Table 2). Therefore, considering a PSQI score of >5 points to indicate poor sleep, 49.2% (*n* = 221) of the PWE cohort were considered “poor sleepers.”

**TABLE 2:** Descriptive statistics of the evaluated scores.

People with epilepsy							General German population			People with migraine		
Score	*N*	Mean	SD	Range	Median	IQR	*N*	Mean	SD	*N*	Mean	SD
**Pittsburgh Sleep Quality Index (PSQI)**												
Total score	449	6.5	3.8	0–19	5	4–9	9284	5	3.4	2389	6.5	3.6
Men	188	6.0	3.7	0–18	5	3–8	4420	4.4	3.0		n.r.	n.r.
Women	261	6.8	3.8	0–19	6	4–9	4864	5.5	3.6		n.r.	n.r.
Subjective sleep quality	449	1.3	.7	0–3	1	1–2		1.1	.7		n.r.	n.r.
Sleep latency	449	1.4	1.0	0–3	1	1–2		1.1	.9		n.r.	n.r.
Sleep duration	449	.6	.9	0–3	0	0–1		.6	.8		n.r.	n.r.
Sleep efficiency	449	.8	1.1	0–3	0	0–1		.7	1.0		n.r.	n.r.
Sleep disturbance	449	1.2	.5	0–3	1	1–1		.6	.6		n.r.	n.r.
Use of sleep medication	449	.2	.7	0–3	0	0–0		.1	.6		n.r.	n.r.
Daytime dysfunction	449	1.1	.8	0–3	1	1–2		.8	.7		n.r.	n.r.
Above the threshold for poor sleep quality (>5 points), % (*n*)	49.2 (221)						35.9 (n.r.)			53.5 (1277)		
**Hospital Anxiety and Depression Scale, German version (HADS)**												
Total score	443	11.4	7.6	0–38	10	5–16	4410	n.r.	n.r.		n.r.	n.r.
Men	186	11.0	7.5	0–32	10	5–16	1929	9.2	6.7		n.r.	n.r.
Women	257	11.6	7.7	0–38	10	6–16	2481	9.7	6.9		n.r.	n.r.
HADS‐A (anxiety) subscore	443	5.9	4.2	0–19	5	3–9	4410	n.r.	n.r.		n.r.	n.r.
Men	186	5.3	4.2	0–17	4	2–8	1929	4.4	3.3		n.r.	n.r.
Women	257	6.4	4.1	0–19	6	3–9	2481	5.0	3.6		n.r.	n.r.
HADS‐D (depression) subscore	443	5.5	4.3	0–20	4	2–8	4410	n.r.	n.r.	2389	4.3	3.6
Men	186	5.7	4.3	0–20	5	2–8	1929	4.8	4.0		n.r.	n.r.
Women	257	5.3	4.3	0–19	4	2–8	2481	4.7	3.9		n.r.	n.r.
Above the threshold for significant anxiety and depression (≥13 points)^c^, % (*n*)	37.4 (168)						28.0^a^/31.5^b^				n.r.	n.r.
Above the threshold for significant anxiety (≥8 points)^d^, % (*n*)	30.5 (137)						18.1^a^/23.2^b^				n.r.	n.r.
Above the threshold for significant depression (≥8 points)^d^, % (*n*)	27.4 (123)						23.9^a^/23.5^b^				n.r.	n.r.
**Quality of Life in Epilepsy – 31 item (QOLIE‐31) inventory**												
Total score	426	62.6	18.3	10.8–97	64.5	51.2–77.2	n.r.					
T‐Score	426	49.8	11.2	18–71	51	43–59	n.r.					

Abbreviations: IQR, interquartile range; n.r., not reported; SD, standard deviation.

^a^ Men.

^b^ Women.

^c^ Cutoff according to Hinz and Brähler.^17^

^d^ Cutoff according to Zigmond and Snaith.^24^

### 3.5 HADS

The total HADS score, an aggregate of the HADS‐D and HADS‐A subscales, was 11.4 points (SD ± 7.6) in the PWE cohort. Women rated numerically higher than men (mean 11.6 [SD ± 7.7] vs 11.0 [SD ± 7.5], *p* = .445). Considering the subscales separately, depression symptoms did not differ significantly by sex, although men scored numerically higher (mean 5.7 [SD ± 4.3] vs 5.3 [SD ± 4.3], *p* = .227). In contrast, women reported significantly greater anxiety than men (mean 6.4 [SD ± 4.1] vs 5.3 [SD ± 4.2], *p* < .001).

### 3.6 QOLIE‐31

In the PWE cohort, the mean total QOLIE‐31 score was 62.6 (SD ± 18.3, range 10.8–97), corresponding to a mean T‐Score of 49.8 (SD ± 11.2, range 18–71). Considering only Item 31 of the QOLIE‐31 instrument, the mean epilepsy‐related QoL score was 66.7 (SD ± 16.2, range 0–100).

### 3.7 Correlation analyses

**FIGURE 2:**
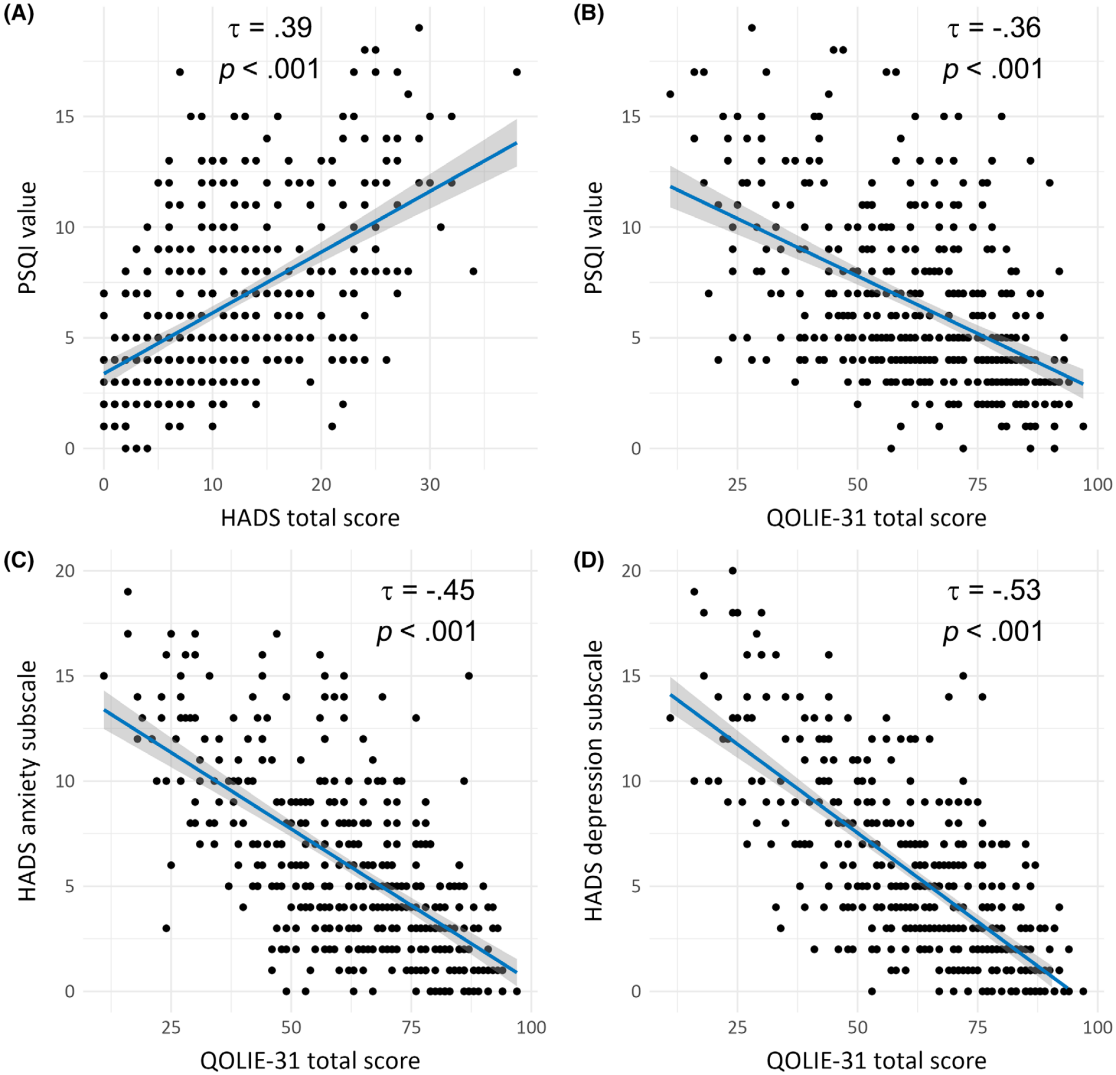
Correlation analysis results between sleep, affective disorders, and quality of life (QoL). (A) Impaired sleep quality (Pittsburgh Sleep Quality Index [PSQI]) was significantly positively correlated with increased affective symptoms (Hospital Anxiety and Depression Scale [HADS]). (B) Sleep quality was significantly negatively correlated with health‐related QoL (Quality of Life in Epilepsy ‐ 31 items [QOLIE‐31]). (C, D) Health‐related QoL (QOLIE‐31) was significantly negatively correlated with both anxiety (HADS‐A) and depression (HADS‐D). All correlations were performed using Kendall's rank correlation method (*τ*).

PSQI correlated positively with HADS total score (*τ* = .39, *p* < .001), that is, worse sleep correlated with higher combined anxiety and depression (Figure 2). A negative correlation of the PSQI with the QOLIE‐31 total score (*τ* = −.36, *p* < .001) was apparent, connecting worse sleep with lower QoL. Both the anxiety and depression subscales of the HADS strongly negatively correlated with the QOLIE‐31 total score (*τ* = −.45 and *τ* = −.53, respectively, both *p*'s < .001).

### 3.8 Univariable analysis of sleep quality (PSQI)

Sociodemographic variables other than sex (see previous text) were associated with PSQI scores in univariable analyses. These included age, where increasing age was associated with worse sleep quality, with the PSQI score increasing by .03 points per year (*p* = .019). Relationship status (*p* = .020) and unemployment (*p* < .001) were also associated with significantly higher PSQI scores, as was having a disability certificate (*p* = .020).

Among the clinical factors, BMI was not significantly associated with worse PSQI scores, although it showed a trend (*p* = .054). The type of epilepsy (frontal lobe vs other focal vs genetic generalized), the epilepsy duration, seizure frequency, or current number of ASM were not significantly associated with PSQI scores in univariable analysis. However, an almost consistent trend toward worse sleep quality with increasing seizure frequency was apparent, with >1 year seizure‐free individuals exhibiting a mean PSQI of 6.18 (SD ± 3.59), compared to 7.79 (SD ± 4.34) for those with daily seizures (Table 3). A similar trend was observed for the number of ASMs: PWE without current ASM use rated lower on the PSQI (6.12 [SD ± 4.33]) than those with at least triple therapy (6.87 [SD ± 4.05]). In addition, using a non‐ASM sleeping medication or sedative was associated with worse sleep on the PSQI (*p* = .002; Table 3).

**TABLE 3:** Univariable analyses of sleep quality (PSQI).

Variable	Category	Value	*p*‐value
Age (estimate)	Years	.03	.**019** ^a^
BMI (estimate)	kg/m^2^	.03	.054^a^
Sex, mean [SD]	Men	6.01 [3.74]	.**009** ^b^
	Women	6.84 [3.79]	
Epilepsy type	Focal: Frontal lobe epilepsy	6.43 [4.59]	.796^b^
	Focal: Other lobes	6.58 [3.75]	
	Genetic generalized	6.35 [3.7]	
	Unclassified	6.39 [3.59]	
Mean duration of epilepsy (estimate)	Years	.003	.667^a^
Seizure frequency	Daily	7.79 [4.34]	.174^b^
	Weekly	7.3 [3.86]	
	Monthly	6.92 [4.08]	
	Once every 6 months	6.07 [3.94]	
	Once per year	6.43 [3.62]	
	No seizures in >1 year	6.18 [3.59]	
Number of anti‐seizure medications (ASMs)	0	6.12 [4.33]	.789^b^
	1	6.34 [3.64]	
	2	6.51 [3.77]	
	≥3	6.87 [4.05]	
Relationship status	Married/in a relationship	6.53 [3.64]	.**020** ^b^
	Divorced/separated	8.62 [4.5]	
	Single/living with relatives	5.87 [3.9]	
	Living alone	6.69 [3.96]	
	Widowed	4 [2.83]	
Occupational situation	Working or vocational training	5.84 [3.34]	**<.001** ^b^
	Not working (Stay‐at‐home/Unemployed/Disability pension/Retired)	7.63 [4.24]	
Disability certificate	No	5.97 [3.46]	.**020** ^b^
	Yes	6.91 [3.97]	
Any sleep‐related medication/sedatives	No	6.26 [3.66]	.**002** ^b^
	Yes	8.03 [4.23]	

*Note*: Bold indicates significance level of *p* < .05.

Abbreviations: BMI, body mass index; SD, standard deviation.

^a^ Univariable ordinal regression.

^b^ Kruskal–Wallis test.

### 3.9 Multivariable analysis of sleep quality (PSQI)

Several variables were independently associated with worse sleep quality (higher PSQI scores) in the multivariable model (Table 4). The model (Akaike information criterion [AIC] = 1960.8) exhibited a significantly better fit than the null model (AIC = 2049.7; *χ* ^2^ = 130.97, degrees of freedom [*df*] = 21, *p* < .001). Overall, the model explained 29% of the variance in PSQI scores (Nagelkerke's pseudo *R* ^2^ = .289).

**TABLE 4:** Multivariable analyses of sleep quality (PSQI) and epilepsy‐associated quality of life (QOLIE‐31).

Variable	Value	OR	95% CI	*p*‐value
**Dependent variable: PSQI**				
Age	Years	1.01	1.00–1.03	.141
BMI	kg/m^2^	1.02	.99–1.06	.187
Sex	Women	2.02	1.36–3.01	.**001**
	Men^a^	Ref.	Ref.	
Relationship status	Divorced/separated	1.22	.51–2.94	.654
	Single/living with relatives	1.13	.64–1.98	.681
	Living alone	1.55	.92–2.61	.096
	Widowed	.09	.02–.46	.**004**
	Not reported	.58	.07–4.95	.618
	Married/in a relationship^a^	Ref.	Ref.	
Occupational situation	Not working (Stay‐at‐home/Unemployed/Disability pension/Retired)	1.74	1.10–2.73	.**017**
	Working or vocational training	Ref.	Ref.	
HADS‐D (depression) subscale, dichotomized	Yes (≥8 points)	2.19	1.36–3.54	.**001**
	No^a^	Ref.	Ref.	
HADS‐A (anxiety) subscale, dichotomized	Yes (≥8 points)	3.79	2.39–6.04	**<.001**
	No^a^	Ref.	Ref.	
Disability certificate	Yes	.88	.59–1.31	.530
	No^a^	Ref.	Ref.	
Any sleep‐related medication/sedatives	Yes	1.28	.73–2.23	.388
	No^a^	Ref.	Ref.	
Seizure frequency	Once per year	1.38	.79–2.42	.262
	Once every 6 months	1.10	.54–2.22	.796
	Monthly	.98	.60–1.62	.947
	Weekly	.89	.46–1.70	.716
	Daily	2.55	1.03–6.30	.**042**
	No seizure in >1 year^a^	Ref.	Ref.	
Epilepsy type	Generalized	.85	.44–1.65	.635
	Focal: Other lobes	.83	.46–1.50	.547
	Focal: Frontal lobe epilepsy	.64	.28–1.43	.273
	Unclassified^a^	Ref.	Ref.	
Observations (*n*)			386	
**Dependent variable: QOLIE‐31**				
Age	Years	.99	.98–1.01	.566
BMI	kg/m^2^	.98	.95–1.02	.340
Sex	Women	.88	.59–1.33	.549
	Men^a^	Ref.	Ref.	
Relationship status	Divorced/separated	.80	.33–1.90	.610
	Single/living with relatives	.91	.50–1.65	.755
	Living alone	.86	.51–1.44	.562
	Widowed	4.28	.89–20.68	.071
	Not reported	2.32	.29–18.37	.424
	Married/in a relationship^a^	Ref.	Ref.	
Occupational situation	Not working (Stay‐at‐home/Unemployed/Disability pension/Retired)	.74	.46–1.21	.236
	Working or vocational training^a^	Ref.	Ref.	
HADS‐D (depression) subscale, dichotomized	Yes (≥8 points)^a^	Ref.	Ref.	
	No	7.91	4.63–13.53	**<.001**
HADS‐A (anxiety) subscale, dichotomized	Yes (≥8 points)^a^	Ref.	Ref.	
	No	4.45	2.71–7.30	**<.001**
Disability certificate	Yes^a^	Ref.	Ref.	
	No	1.53	1.01–2.32	.**043**
Any sleep‐related medication/sedative	Yes^a^	Ref.	Ref.	
	No	1.49	.83–2.67	.184
Seizure frequency	Once per year	.65	.35–1.20	.170
	Once every 6 months	.48	.24–.94	**.032**
	Monthly	.25	.15–.44	**<.001**
	Weekly	.21	.10–.42	**<.001**
	Daily	.21	.08–.55	.**002**
	No seizure in >1 year^a^	Ref.	Ref.	
Epilepsy type	Genetic generalized	1.81	.92–3.57	.086
	Focal: Other lobes	1.44	.80–2.60	.224
	Focal: Frontal lobe epilepsy	1.63	.68–3.89	.273
	Unclassified^a^	Ref.	Ref.	
PSQI score	Above the threshold for poor sleep quality (>5 points)^a^	Ref.	Ref.	
	Below the threshold	1.79	1.20–2.68	.**005**
Observations (*n*)			365	

*Note*: Bold indicates significance level of *p* < .05.

Abbreviations: 95% CI, 95% confidence interval; BMI, body mass index; HADS, Hospital Anxiety and Depression Scale; OR, odds ratio; PSQI, Pittsburgh Sleep Quality Index; QOLIE‐31, Quality of Life in Epilepsy – 31 items; SWS, Seizure Worry Scale.

^a^ Reference category.

Women with epilepsy had twofold greater odds of worse sleep quality (odds ratio [OR] = 2.02, 95% confidence interval [95% CI] = 1.36–3.01, *p* = .001). In addition, unemployment was associated with a 74% greater odds of worse sleep quality (OR = 1.74, 95% CI = 1.10–2.73, *p* = .017). Higher HADS‐D and HADS‐A scores were both independently associated with worse sleep quality. Scoring positive for anxiety on the HADS‐A was associated with an almost fourfold higher odds of worse sleep (OR 3.79, 95% CI = 2.39–6.04, *p* < .001) and similarly, having a positive screening result for depression on the HADS‐D was associated with a more than twofold increased odds for worse sleep (OR 2.19, 95% CI = 1.36–3.54, *p* = .001). Regarding marital status, widowers had 91% lower odds of worse sleep quality (OR = .09, 95% CI = .02–.46, *p* = .004), although this was potentially biased by the very small number of widowers in the PWE cohort (*n* = 5).

Besides these demographic and HADS variables, only one clinical variable was independently associated with worse sleep quality. Participants with daily seizures had a more than twofold greater odds for worse sleep quality than those who were seizure‐free for >1 year (OR = 2.55, 95% CI = 1.03–6.30, *p* = .042), whereas no significant difference emerged for those experiencing seizures less frequently than daily. Age, BMI, epilepsy syndrome, or use of sleep medication/sedatives did not predict worse sleep quality. Having a disability card also did not emerge as independent predictors of higher PSQI scores.

### 3.10 Multivariable analysis of epilepsy‐related quality of life (QOLIE‐31)

In the analysis of factors associated with epilepsy‐related QoL, higher HADS‐A and HADS‐D scores emerged as significant independent correlates of poorer QoL. Participants screening negative for anxiety demonstrated a more than fourfold increase in the likelihood of reporting better QoL (OR = 4.45, 95% CI = 2.71–7.30, *p* < .001), whereas those screening negative for depression had a nearly eightfold higher odds (OR = 7.91, 95% CI = 4.63–13.53, *p* < .001). Individuals experiencing seizures at least once every 6 months were significantly more likely to report lower epilepsy‐specific QoL compared to seizure‐free participants (OR = .48, 95% CI = .24–.94, *p* = .032), with a progressively decreasing trend in QoL observed among those with more frequent seizures. The most severely affected subgroup—those with daily seizures—had markedly lower odds of favorable QoL (OR = .21, 95% CI = .08–.55, *p* = .002; Table 4). Absence of a disability card was also associated with higher odds of better QoL (OR = 1.53, 95% CI = 1.01–2.32, *p* = .043). Still, PSQI scores remained independently linked to QoL, with participants reporting no sleep disturbances showing a 79% increase in the odds of better QoL (OR = 1.79, 95% CI = 1.20–2.68, *p* = .005).

The model (AIC = 2912.2) exhibited a significantly better fit than the null model (AIC = 3171.7; *χ* ^2^ = 303.53, df = 22, *p* < .0001). Overall, the model explained 56% of the variance in QOLIE‐31 (QoL) scores (Nagelkerke's pseudo *R* ^2^ = .564).

### 3.11 Comparison to general German population and those with chronic migraine

#### 3.11.1 PSQI

Overall sleep quality was significantly worse in the PWE cohort than in the general German population (*p* < .001), where the mean was below the PSQI cutoff for “poor sleep” (5, SD ± 3.4) and only 35.9% are “poor sleepers.” Worse overall sleep quality than in the general population was also observed separately for men and women with epilepsy (both *p* < .001) (Table 2). Among the PSQI subscales, the largest differences were in higher sleep disturbance (mean 1.2 [SD ± .5] vs .6 [SD ± .6], *p* < .001) and more severe daytime dysfunction (mean 1.1 [SD ± .8] vs .8 [SD ± .7], *p* < .001) in PWE. Sleep quality (*p* < .001), sleep latency (*p* < .001), and sleep efficiency (*p* = .040) were also worse in PWE. The use of sleep medication was also significantly more frequent in PWE (*p* = .001), whereas sleep duration did not differ significantly between the PWE and the general population (*p* = 1.000).

The mean PSQI score in PWE was identical to a large cohort of 2389 individuals with chronic migraine^18^ (both 6.5, *p* = 1.000), another common chronic neurologic disorder that often affects sleep.

#### 3.11.2 HADS

Total HADS scores were significantly higher in the PWE cohort than in the general German population for both men (mean 9.7, SD ± 6.9, *p* < .001) and women (mean 9.2, SD ± 6.7, *p* = .001). In addition, significantly more depressive symptoms were reported in the PWE cohort than in the general German population for both men (mean 4.8, SD ± 4.0, *p* = .004) and women (mean 4.7, SD ± 3.9, *p* = .020). Depressive symptoms were also more severe in PWE than in individuals with chronic migraine (mean 5.5 [SD ± 4.3] vs 4.3 [SD ± 3.6] *p* < .001). Moreover, anxiety was significantly greater in the PWE cohort than in the general German population for both men (mean 4.4, SD ± 3.3, *p* = .001) and women (mean 5.0, SD ± 3.6, *p* < .001).

## 4 DISCUSSION

This study investigated predictors of worse sleep quality in a large multicenter cohort of individuals with predominantly pharmacoresistant epilepsy and the effect of sleep problems on epilepsy‐related QoL.

Its key finding was a significant link between comorbid depression and anxiety and poorer sleep quality in PWE. Worse sleep quality was also independently associated with worse QoL. Overall, sleep quality of PWE was significantly worse than that of the general German population.

### 4.1 Variables associated with reduced sleep quality in PWE

Among demographic variables, female sex and older age were associated with worse sleep quality. We also observed a trend toward poorer sleep quality with increasing seizure frequency. Although this trend was not statistically significant in univariable analysis, the multivariable analysis revealed that individuals with the highest seizure burden (daily seizures) had significantly poorer sleep quality compared to seizure‐free individuals. Results from previous studies suggest that this might be explained by fragmented sleep or an overall worse post‐ictal condition.^25^ However, our study design precluded a more detailed analysis of specific mechanisms, as polysomnographic sleep stage analysis or the number of specifically nocturnal seizures was not available to us. Epilepsy type (frontal vs other focal vs genetic generalized etiologies) and duration were not associated independently with sleep quality. This may suggest that seizure frequency itself has a greater impact on sleep quality than the predominant seizure type.

It is well known that sleep and epilepsy have a complex and reciprocal relationship,^3^ and several aspects of epilepsy are commonly presumed to directly prevent good sleep.^3^, ^7^ Our results suggest that common comorbidities of epilepsy, such as affective disorders, are strongly associated with reduced sleep quality in PWE in addition to seizure frequency and ASM use. Among the variables examined, HADS‐A (anxiety) and HADS‐D (depression) scores showed the strongest associations with poorer sleep quality. It is well‐known that affective disorders principally disturb sleep, and a reciprocal relationship exists, not unlike the one between seizures and sleep.^26^, ^27^ Our results suggest that depression and anxiety should be increasingly considered when evaluating and addressing reduced sleep quality in PWE. However, CIs were wide, especially for anxiety (OR 3.79, 95% CI 2.39–6.04), suggesting that the effect of anxiety on sleep differs even though the overall association is strong. This is also reflected in the comparatively low amount of variance (29%) explained by our regression model, suggesting that there are other factors decreasing sleep quality in PWE not accounted for in this analysis. One large meta‐analysis also found higher PSQI scores in PWE but could not elucidate risk factors, potentially due to heterogeneity among studies.^10^ Our study confirms similar findings on the link between sleep quality and epilepsy from earlier studies on PWE in China, the United States, and Austria.^6^, ^28^, ^29^

Use of sleep‐related medication was associated with worse sleep quality in univariable but not multivariable analysis in our cohort. Barbiturate ASMs and chronic benzodiazepine use was limited in our cohort (2% and 2.7%, respectively). Previous research has shown that certain ASMs, such as lamotrigine and phenobarbital, are associated with poorer sleep quality, whereas others like perampanel may have more favorable effects on sleep parameters in people with epilepsy.^30^ The impact of polytherapy and specific ASM profiles on sleep warrants further investigation. In addition, the potential influence of co‐prescription of antidepressants and antipsychotics on sleep quality in PWE should be evaluated systematically in future studies. Unfortunately, affective disorders are still insufficiently treated in PWE despite advances.^31^ This aligns with our findings, which demonstrated a discrepancy between the proportion of patients reporting depressive symptoms (27.4%) and those receiving antidepressant treatment (8%); however, given the limitations of self‐reported data vs formal diagnostic assessment, this gap should be interpreted with caution.

Notably, increased body weight was not significantly associated with worse PSQI outcomes (*p* = .187). The prevalence of overweight (BMI ≥25 and <30: 32.3%) and obesity (BMI ≥30: 13.5%) was slightly lower than in the general German population (36% and 16.7%, respectively), although not to a degree suggesting systematic underrepresentation.^32^ It is possible that body weight may not exert the same influence on sleep quality in individuals with a severe chronic condition such as epilepsy—particularly in our sample drawn from tertiary epilepsy centers—as it does in the general population.

### 4.2 PSQI and domain‐specific sleep deficits in PWE

On average, sleep quality of PWE in our cohort was significantly impaired, with a mean PSQI of 6.5 (SD ± 3.8) points above the cutoff (>5 points). Mean PSQI in a recent meta‐analysis was just below the cutoff (4.94 points, SD ± 3.03), probably reflecting inter‐study heterogeneity of clinical severity.^10^ Our finding of worse sleep quality in PWE compared to the general population is in line with findings of a previous study that found a twofold greater prevalence of sleep disturbance in individuals with partial epilepsy.^5^ Another recent study that collected data via a multinational European online survey also demonstrated higher PSQI scores in PWE.^30^ In that study, 80% of PWE had a PSQI score above the cutoff of 5 points, more than in our study (49.2%).

Considering the PSQI subscales, it is interesting that the largest absolute differences from the general population were higher sleep disturbance and more severe daytime dysfunction in PWE, whereas sleep efficiency and sleep latency were also worse. This supports earlier findings comparing PSQI subscales between PWE and the general population.^30^ This observation corresponds to the notion of reduced sleep efficiency and altered sleep structure in PWE.^25^ However, we caution that we could not disentangle the specific contributions of seizures and potential unwanted effects of ASMs from our data. It should again be noted that affective disorders also disturb sleep and reduce daytime functioning.

### 4.3 Sleep quality and epilepsy‐related QoL

It was shown previously that seizures impair QoL, as measured by the 36‐item Short Form health survey.^5^ In addition, worse PSQI scores correlated negatively with generic QoL (12‐item Short Form health survey) and depression in a recent European study,^30^ similar to another recent study in India^33^ and an older study from the United States.^6^ Our study confirmed this association for epilepsy‐related QoL (QOLIE‐31). Another study also found higher depressive symptoms in PWE with elevated PSQI scores.^34^ A previous study in the UK found anxiety to be a predictor of worse QoL in PWE.^35^ We confirmed this finding using the HADS, a generic depression/anxiety scale. We found sleep quality to be similar in PWE and individuals with chronic migraine, which severely affects sleep and where nocturnal attacks are also associated with increased disability and lower QoL.^36^ However, we could not account for relative disease burden due to inter‐study comparison of aggregate data, limiting the explanatory power of this comparison compared to a matched case–control analysis.

Overall, QoL in our PWE cohort corresponded to QoL in the cohort used to develop the QOLIE‐31 instrument (mean T score of 49.8 in our cohort). Because our data were collected at tertiary epilepsy centers, pharmacoresistant epilepsy was more common than overall in our PWE cohort. QoL was more significantly affected by seizure severity, reflecting the QOLIE‐31 being an epilepsy‐specific QoL instrument.

### 4.4 Limitations

Several limitations exist with this study. Several clinical variables potentially influencing the role of an individual's epilepsy on sleep quality could not be captured in our cross‐sectional questionnaire analysis. This includes, for example, objective sleep stages and nocturnal seizure frequency, potentially biasing and reducing the actual influence of epilepsy in our analysis. Further large‐scale video‐EEG monitoring‐based studies are desirable to fill this gap.^37^ The broad age range in our cohort may introduce heterogeneity due to certain uncaptured age‐related sleep/psychiatric disorders. Although age was included as a covariate in the multivariable analyses to account for some of this variance, further stratification by age groups was not pursued due to the lack of clear cutoffs and the associated reduction in statistical power. The relationship/marital status variable includes several categories with limited sample sizes, which constrains the statistical power of findings related to this variable. However, the more granular model demonstrated a statistically superior fit compared to a slightly more parsimonious alternative; therefore, we opted to retain the more detailed specification.

Although we captured a wide range of clinical epilepsy severity, from seizure‐free patients to those with ongoing daily seizures and polytherapy, our data still stem from specialized epilepsy centers, potentially reducing the external validity to PWE overall. Comparisons to the German general population were only possible using previously published aggregate data from other studies and not on a matched case–control basis; thus we could not account for potentially important confounders such as overall disease burden, thereby limiting the explanatory scope of these comparisons. Because of limitations in the questionnaire design, we were unable to specifically assess the impact of reduced sleep quality on mood and affective symptoms, which limited our ability to perform a fully reciprocal analysis.

## 5 CONCLUSIONS

Our findings suggest that affective disorders are significantly associated with poorer sleep quality in PWE, particularly among those with difficult‐to‐treat or pharmacoresistant forms. Despite growing scientific and clinical awareness, affective comorbidities remain underrecognized and undertreated in this population. Given the observed association between poor sleep quality and diminished QoL, clinicians may consider routinely assessing and addressing both affective symptoms and sleep disturbances as part of comprehensive care to improve treatment outcomes and QoL.

## AUTHOR CONTRIBUTIONS

**Conceptualization:** Johann Philipp Zöllner and Adam Strzelczyk. **Data curation:** Johann Philipp Zöllner, Laurent Willems, and Adam Strzelczyk. **Formal analysis:** Johann Philipp Zöllner. **Funding acquisition:** Adam Strzelczyk. **Investigation:** Johann Philipp Zöllner, Catrin Mann, Laurent Willems, and Adam Strzelczyk. **Methodology:** Johann Philipp Zöllner and Adam Strzelczyk. **Project administration:** Johann Philipp Zöllner, Catrin Mann, Laurent Willems, and Adam Strzelczyk. **Resources:** Johann Philipp Zöllner, Felix Rosenow, and Adam Strzelczyk. **Software:** Johann Philipp Zöllner and Adam Strzelczyk. **Supervision:** Felix Rosenow and Adam Strzelczyk. **Validation:** Johann Philipp Zöllner, Felix Rosenow, and Adam Strzelczyk. **Visualization:** Johann Philipp Zöllner and Adam Strzelczyk. **Writing ‐ original draft:** Johann Philipp Zöllner, Catrin Mann, Laurent Willems, Felix Rosenow, and Adam Strzelczyk. **Writing ‐ review & editing:** Johann Philipp Zöllner, Catrin Mann, Laurent Willems, Felix von Podewils, Lisa Langenbruch, Laura Bierhansl, Susanne Knake, Katja Menzler, Juliane Schulz, Bernadette Gaida, Felix Rosenow, and Adam Strzelczyk.

## FUNDING INFORMATION

This study was supported by the state of Hesse, Germany, via the LOEWE grant for the Center for Personalized, Translational Epilepsy Research (CePTER) at the Goethe University, Frankfurt am Main, Germany.

## CONFLICT OF INTEREST STATEMENT

J.P. Zöllner has received support in the form of speakers' honoraria and travel reimbursements from Jazz Pharmaceuticals and Danone, outside the submitted work; he was supported by a research grant from the Vereinigung von Freunden und Förderern (VFF) – Dr. Reiss Stiftung as well as research support from the Chaja‐Stiftung, Desitin Arzneimittel, Dr. Schär Deutschland GmbH, Nutricia Milupa GmbH, and Vitaflo Deutschland GmbH, outside the submitted work. C. Mann reports speakers' honoraria and travel support from UCB Pharma, outside the submitted work. C. Mann was supported by the Emmy Klieneberger Nobel grant for women scientists from the Medical Faculty of the Goethe University, Frankfurt. F. von Podewils received personal fees as a speaker or for serving on advisory boards from Angelini Pharma, Arvelle, Bial, Desitin Arzneimittel, Eisai, Jazz Pharmaceuticals, UCB Pharma, and Zogenix. L. Langenbruch received speakers' honoraria from Eisai and GW Pharmaceuticals. L. Bierhansl reports travel supports from Angelini Pharma and Jazz Pharmaceuticals, outside the submitted work. S. Knake received speakers' honoraria from Bial, Desitin Arzneimittel, Eisai, Jazz Pharmaceuticals, Merck Serono, and UCB Pharma. K. Menzler has received speakers' honoraria from UCB Pharma, Eisai, Bial, and Jazz Pharmaceuticals. B. Gaida reports speakers' honoraria from UCB Pharma. F. Rosenow reports honoraria for scientific advice and as speaker from Angelini Pharma, Eisai, Jazz Pharmaceuticals, Stoke Therapeutics, Takeda, and UCB Pharma. Research support: European Union (EU‐FP7), German Research Foundation (DFG), Federal State of Hesse, Germany, Detlev Wrobel Fonds for Epilepsy Research, Dr. Reiss‐Stiftung, Dr. Senckenbergische‐Stiftung, Kassel‐Stiftung, Ernst Max von Grunelius‐Stiftung, Chaja‐Stiftung, Desitin Arzneimittel, Dr. Schär Deutschland GmbH, Nutricia Milupa GmbH, and Vitaflo Deutschland GmbH. A. Strzelczyk reports personal fees or research funding from Angelini Pharma, Biocodex, Desitin Arzneimittel, Eisai, Jazz Pharmaceuticals, Longboard, Neuraxpharm, Stoke Therapeutics, Takeda, UCB Pharma, and UNEEG. L.M. Willems and J. Schulz report no conflicts of interest.

## ETHICS APPROVAL

This study was approved by the ethics committee of the Goethe‐University Frankfurt (reference number 19‐440). We confirm that we have read the Journal's position on issues involved in ethical publication and affirm that this report is consistent with those guidelines.

## PATIENT CONSENT

All participating individuals were included after written informed consent.

## TRIAL REGISTRATION

German Clinical Trials Register (DRKS00022024; Universal Trial Number: U1111‐1252‐5331).

## Data Availability

The datasets generated and/or analyzed in this study are not publicly available due to national data protection laws but are available from the corresponding author upon reasonable request.
